# Enhancing potential impact of hospital discharge interventions for patients with COPD: a qualitative systematic review

**DOI:** 10.1186/s12913-023-09712-0

**Published:** 2023-06-22

**Authors:** Torbjørn Nygård, David Wright, Hamde Nazar, Svein Haavik

**Affiliations:** 1 Department of Clinical Science, University of Bergen, P.O. box 7804, 5020 Bergen, Norway; 2 School of Healthcare, University of Leicester, Leicester, UK; 3 School of Pharmacy, Newcastle University, Newcastle Upon Tyne, UK

**Keywords:** Chronic obstructive pulmonary disease, Systematic review, Qualitative research, Implementation science, Health services

## Abstract

**Background:**

Patients with chronic obstructive pulmonary disease (COPD) are frequently readmitted to hospital resulting in avoidable healthcare costs. Many different interventions designed to reduce hospital readmissions are reported with limited evidence for effectiveness. Greater insight into how interventions could be better designed to improve patient outcomes has been recommended.

**Aim:**

To identify areas for optimisation within previously reported interventions provided to reduce COPD rehospitalisation to improve future intervention development.

**Methods:**

A systematic review was conducted by searching Medline, Embase, CINAHL, PsycINFO, and CENTRAL in June 2022. Inclusion criteria were interventions provided to patients with COPD in the transition from hospital to home or community. Exclusion criteria were lack of empirical qualitative results, reviews, drug trials, and protocols. Study quality was assessed using the Critical Appraisal Skills Programme tool and results were synthesised thematically.

**Results:**

A total of 2,962 studies were screened and nine studies included. Patients with COPD experience difficulties when transitioning from hospital to home. It is therefore important for interventions to facilitate a smooth transition process and give appropriate follow-up post-discharge. Additionally, interventions should be tailored for each patient, especially regarding information provided.

**Conclusion:**

Very few studies specifically consider processes underpinning COPD discharge intervention implementation. There is a need to recognise that the transition itself creates problems, which require addressing, before introducing any new intervention. Patients report a preference for interventions to be individually adapted—in particular the provision of patient information. Whilst many intervention aspects were well received, feasibility testing may have enhanced acceptability. Patient and public involvement may address many of these concerns and greater use of process evaluations should enable researchers to learn from each other’s experiences.

**Trial registration:**

The review was registered in PROSPERO with registration number CRD42022339523.

**Supplementary Information:**

The online version contains supplementary material available at 10.1186/s12913-023-09712-0.

## Introduction

Patients with chronic obstructive pulmonary disease (COPD) suffer from the revolving door syndrome, in which the patients are frequently readmitted to hospital [1, 2]. Hospitalisations are frequently caused by acute worsening of respiratory symptoms [1, 3]. This contributes not only towards burden for the patients themselves [4], but also to high costs for hospitals and health care systems [5]. It is estimated that the annual cost of treating patients with COPD in the European Union is 38.6 billion euros [1]. As such, it is a priority to reduce hospital readmissions, which could have been avoided by preventative measures, to improve health and care for patients with COPD and to reduce the economic burden.

There are many different interventions reported for patients with COPD in order to reduce hospital readmissions. This includes interventions such as pulmonary rehabilitation, self-management, medicines support, hospital-at-home support services, and telemonitoring. Pulmonary rehabilitation involves exercise, education, and behaviour change to reduce COPD symptoms and improve quality of life [6]. Self-management interventions aim to empower patients and have them develop skills to better manage their disease [7]. Medicines support interventions are designed to improve medicines prescribing and/or medication adherence [8–10]. Hospital-at-home interventions involves moving care from the hospital to the patients’ home to reduce costs and increase patient satisfaction and quality of life [11]. Lastly, telemonitoring is used to monitor patients’ health state at home using technology, in which early detection of disease deterioration is made possible [12]. All of these interventions are complex healthcare interventions and thus require greater consideration regarding design and evaluation [13].

Several systematic reviews have been conducted which investigated effectiveness of different intervention approaches for patients with COPD [14–19]. These studies have found potentially effective interventions, but issues regarding heterogeneity of included studies mean findings are inconclusive [14, 15]. This heterogeneity is especially common in studies about self-management strategies, due to the wide range in focus and delivery of such interventions [16]. Additionally, the systematic reviews report about the low quality of evidence and a need for evaluation of intervention duration and components [17, 18]. This is further underpinned by previously conducted research in which authors recommend improvement to interventions to enhance effectiveness and likelihood of trial success [9, 20–22].

The Medical Research Council’s guidance for developing and evaluating complex interventions recommends that patients are involved in the design and theory is used to inform construction. The intervention should be feasibility tested to optimise its effectiveness and ascertain the best research design before trials are undertaken. Additionally, at both feasibility and definitive trial stages the guidance articulates the need for a better understanding of which parts of interventions have an effect—not only focusing on efficacy or effectiveness—and how to optimise intervention delivery [13]. It is not only important to understand which interventions are effective, but also why they are effective in different contexts and what could influence this effectiveness.

Process evaluations are required to identify intervention fidelity (how well it was delivered), dose (how much of the total intervention was delivered), reach (what proportion of the target audience received it), and mechanism of action (how it actually works). Furthermore, intervention-bundles should only include elements that are effective and fully optimised to maximise cost-effectiveness [13].

The results from process evaluation for all interventions to prevent COPD hospital readmissions have not been combined to identify common themes, which could be used to improve generic intervention effectiveness. Thus, the aim of this paper is to identify areas which could be enhanced to improve the effectiveness of interventions provided to patients with COPD and use these to inform future intervention design. By investigating previous interventions and the qualitative data from the studies undertaken, we can then understand which elements are suitable for a complex health care intervention—as a theoretical basis.

## Methods

This systematic review was registered in the PROSPERO database with registration number CRD42022339523. An unpublished protocol was developed prior to conducting the review. The protocol was updated after initial searches and eligibility evaluations to include better adapted search terms and eligibility criteria. The Cochrane Handbook for Systematic Reviews of Interventions was used as a guide for this review and the reporting was informed by the PRISMA 2020 checklist [23, 24]. The completed PRISMA 2020 checklist is provided in the supplemental material (see Additional file 1).

### Search strategy

The search included Medline/PubMed, Embase, CINAHL, and PsycINFO as databases. The Cochrane Central Register of Controlled Trials (CENTRAL) was used to retrieve trials. The searches included articles up until June 2022 written in English or Scandinavian language. The search strategy was developed by reviewing systematic reviews and meta-analyses with similar scopes to our review [9, 19, 21, 25–30]. Search terms were grouped by participants, interest, and context. Both free text searches and searches using keywords were made. The search strategy was piloted by reviewing 10% of the retrieved reports, in which an additional eligibility criterium requiring qualitative data was added to adjust the number of eligible studies. A detailed description of the search method is given in the supplemental material (see Additional file 2).

### Eligibility criteria

Studies were included if they involved interventions provided to patients with COPD during the transition from a hospital setting to either primary health care, home, or community. The intervention could be provided either before or after hospital discharge. For studies to be eligible for inclusion, they needed to report empirical qualitative data.

Only published peer-reviewed articles including qualitative empirical data relating to COPD interventions to prevent rehospitalisation were included. Review articles, abstracts, posters, and protocols were excluded. The eligibility evaluation was undertaken in two separate steps by TN and LE independently using the Rayyan web application [31]. The first step of the screening process was undertaken by reviewing titles and abstracts. In the second step, full text articles of potentially eligible studies were reviewed to determine final inclusion. Any discrepancies were discussed after each screening process until a consensus was reached. If any discrepancies remained unresolved, a third member from the author team (DW) was included in the discussion process.

### Data extraction and quality assessment

Data extraction from eligible studies was undertaken by TN, managed using Microsoft Excel (Microsoft Corporation, 2022), and a sample was reviewed by DW for accuracy. Study characteristics, intervention related themes, and verbatim textual data were collated. The template for intervention description and replication (TIDieR) checklist and guide was used to collect additional data regarding the intervention and its contextual factors [32]. In this step, further searches were made using Google search engine and Google Scholar (Google LLC, 2022) to detect similar papers which provided more data on intervention details.

The quality assessment of methodology and bias in individual studies was evaluated using the Critical Appraisal Skills Programme (CASP) checklist [33]. The CASP checklist was chosen because of our focus on qualitative data, as it is a recognised assessment tool for qualitative studies. The initial quality assessment was undertaken by TN. All evaluations were reviewed by HN to check for agreement.

### Data analysis and synthesis

A thematic synthesis was undertaken to analyse and synthesise the data similar to the method described by Thomas and Harden [34]. We extracted verbatim textual data from the participants (both patients and healthcare professionals) in each included study. Data on comments regarding research methods were collected as well as comments about interventions. Simultaneously, descriptive themes were extracted from each study (i.e., authors’ views and interpretations of the researched phenomenon). If the studies did not report any descriptive themes, then an inductive coding approach was used to identify the descriptive themes. Based on the extracted data, data condensation was undertaken by reformatting themes and combining similar descriptive themes. Then the descriptive themes were organised and sorted into analytical themes based on similarity of concepts. Finally, the analytical themes were narratively described by comparing and reflecting over the data collected.

## Results

**Fig. 1:**
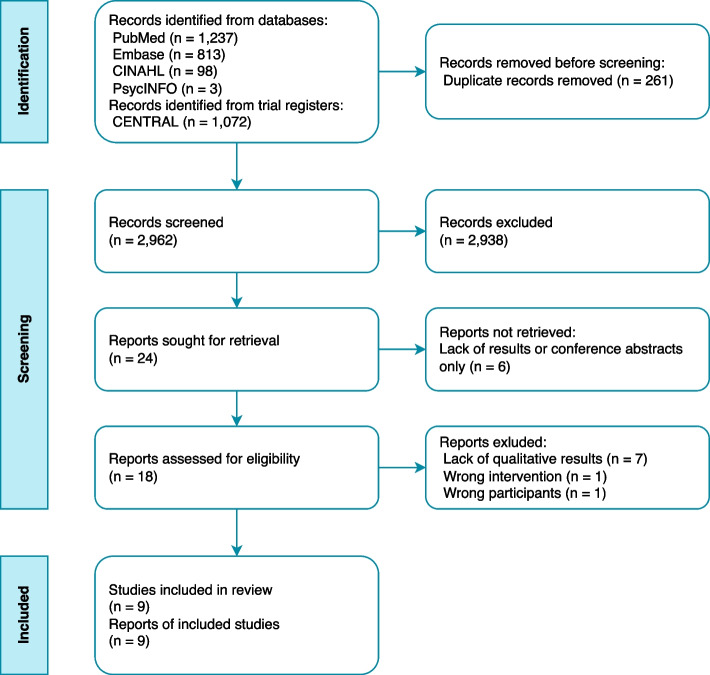
Identification and screening process of the systematic search based on the PRISMA-2020 diagram [24]

A total of 3,223 studies and trials were retrieved from the search. Titles and abstracts were screened after removing duplicates. We included 24 studies from the initial screening and nine articles from the full report screening (Fig. 1). The two reviewers (TN, LE) disagreed on 28 of the initial records, but consensus was reached after brief discussions. A third researcher (DW) was introduced after the screening process to discuss the inclusion of two of the records. Seven studies were excluded at full text review due to their lack of qualitative results [35–41]. Furthermore, one study was removed because there was no discharge process from hospital [42], and one study was removed because patients with COPD only represented a small proportion of the population of interest in the study [43].

### Study characteristics

A summary of the study characteristics and their interventions is given in Table 1. Further details about the interventions and contexts collected through the TIDieR checklist are provided in the supplemental material (see Additional file 3). Most of the studies included were from the UK (*n* = 5). Four of the included studies were randomised controlled trials (RCTs) [44–47], in which two of these had a mixed-methods approach [45, 46]; four of the studies were qualitative studies [48–51]; and one study was a mixed-methods pre-post study [52]. Griffiths and colleagues had included both patients with COPD and patients with congestive heart failure (CHF), and thus only data from patients with COPD were included for analysis [49].

**Table 1:** Characteristics of included studies

Reference (Year)	Country	Study design	Participants (N)	Qualitative sample size	Qualitative data collection method	Qualitative data analysis	Intervention	Context
**Broadbent**[44]** (2018)**	New Zealand	RCT pilot	Patients with COPD (52)	Intervention group: 25	Interviews	Inductive thematic analysis	Home-based, post-discharge robotic telehealth care	Mostly housebound participants from geographically rural locations with poor social support were included. Study took place in South Auckland, where there is a large population of Māori, Pacific Islanders, immigrants, and people with low socioeconomic status
**Buckingham**[45]** (2018)**	UK	Mixed-method feasibility pilot RCT	Patients with severe COPD (19)	8 patients (3 with carers) and 28 social or health care personnel	Individual and group interviews	Framework analysis	Nurse-led, post-discharge care assessment, incl. telephone check-ups	Participants were included from two hospitals in Scotland. Most of the participants were recruited from a tertiary centre, which focuses on innovative care. Participants from the other hospital had similar profile of needs/actions
**Clarke**[48]** (2010)**	UK	Qualitative interview study	Patients with COPD (23)	Intervention group: 14 No intervention: 9	Semi-structured interviews	Grounded theory	Nurse-led early discharge with home-based visits, incl. clinical assessment and checking medicine-use	Participants were recruited in an economically deprived inner-city borough in England
**Cox**[46]** (2018)**	UK	Mixed-method feasibility pilot RCT	Patients with COPD (58)	Intervention group: 27 Staff: 11 No intervention: 2	Semi-structured interviews	Framework analysis	Early pulmonary rehabilitation provided in hospital or at home	Patients were recruited from two centres; both of which are large teaching hospitals
**Griffiths**[49]** (2021)**	Canada	Qualitative interview study	Patients with COPD or CHF and/or their caregivers (16)	8 patients and 8 caregivers	Semi-structured interviews	Directed content analysis	Discharge summary program and physician post-discharge follow-up	Permanent residents have insurance covering physician and hospital services. All primary care physicians in the study are family physicians. Recruitment was undertaken in three acute care or rehabilitation hospitals in two cities in Ontario
**Morton**[52]** (2019)**	UK	Mixed-method controlled pre-post study	Patients with COPD	Not reported	Document analysis, non-participant observation of patient care, in-depth interviews	Inductive and deductive cross-case thematic analysis	Care bundle program and discharge summary	Qualitative data was collected from six acute hospitals in England and Wales; four of which were implementation sites and two were comparator sites. Some of the comparator sites used checklists similar to the intervention as part of usual care. Difference in populations identified through varying COPD readmission rates. Pressure around patient numbers, staffing, and resources in the NHS
**Orme**[47]** (2018)**	UK	Feasibility RCT	Patients with COPD (17)	8 in education and feedback group, 3 in education only group, 13 drop-out interviews	Semi-structured interviews	Constant comparative analysis	Patient education and telemonitoring	Recruitment for the study was undertaken in Glenfield Hospital, which is a university hospital in Leicester
**Utens**[50]** (2013)**	Netherlands	Qualitative results from RCT	Patients with COPD (139)	49 of usual hospital care group and 56 of early assisted discharge group	Two open-ended qualitative questions in a mixed-methods questionnaire	Deductive content analysis	Nurse-led, early discharge hospital-at-home support service	Study included five hospitals and three home care organisations. Different issues arose related to coordination of logistics, indicating differences between the home care organisations
**Wang**[51]** (2012)**	Norway	Qualitative interview study	Patients with COPD (9)	6 receiving intervention, 3 from usual care	Semi-structured interviews	Systematic text condensation	Nurse-led, early discharge hospital-at-home support service	Participants were recruited from Akershus university hospital

*RCT* randomised controlled trial, *COPD* Chronic Obstructive Pulmonary Disease, *CHF* Congestive Heart Failure, *NHS* National Health Service

### Quality assessment of studies

The results from the quality assessment using the CASP tool are provided in Table 2 [33]. The overall value of the included studies was moderate to high. All the qualitative studies had more information available to properly assess study quality. Followingly, the RCT studies had varying levels of methodological quality due to missing elements or unclear reporting.

**Table 2:** Quality assessment of studies using the Critical Appraisal Skills Programme checklist [33]

	**1**	**2**	**3**	**4**	**5**	**6**	**7**	**8**	**9**	**10**
**Broadbent **[44]	Yes	No	?	Yes	?	?	Yes	?	No	Low
**Buckingham **[45]	Yes	Yes	Yes	Yes	Yes	No	Yes	Yes	Yes	Moderate
**Clarke **[48]	Yes	Yes	Yes	Yes	Yes	Yes	Yes	Yes	Yes	High
**Cox **[46]	Yes	Yes	Yes	Yes	Yes	Yes	Yes	Yes	Yes	Very high
**Griffiths **[49]	Yes	Yes	Yes	Yes	Yes	No	Yes	Yes	Yes	High
**Morton **[52]	Yes	Yes	Yes	Yes	Yes	No	Yes	Yes	Yes	High
**Orme **[47]	Yes	Yes	Yes	Yes	Yes	No	Yes	?	Yes	Moderate
**Utens **[50]	Yes	Yes	No	Yes	No	?	Yes	Yes	Yes	Moderate
**Wang **[51]	Yes	Yes	Yes	Yes	Yes	No	Yes	Yes	Yes	Moderate

### Thematic synthesis

**Fig. 2:**
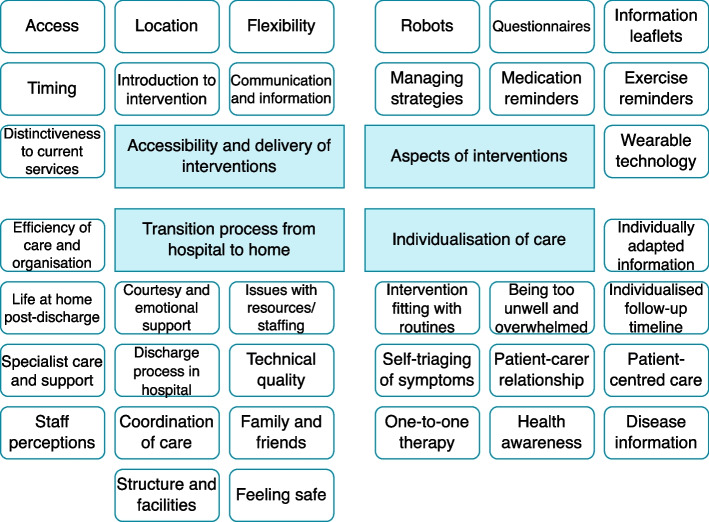
Descriptive and analytical themes from the thematic synthesis. Items with rounded corners are descriptive themes and items with sharp corners are analytical themes

Forty-one themes were extracted from the included studies, in which similar themes were combined for a total of 36 descriptive themes. Minor formatting changes were made to some of the descriptive themes to reflect the formatting of the other themes. The descriptive themes were then combined and sorted into four analytical themes (Fig. 2). The verbatim text extracts are provided in the supplemental material (see Additional file 4).

#### Analytical theme 1: Accessibility and delivery of interventions

Introducing the intervention (i.e., pulmonary rehabilitation) to patients resulted in them experiencing benefits and possible enjoyment of the exercises [46]. The intervention provided in one study was inaccessible to some patients because they were admitted to a different hospital ward than the pulmonary ward—where the intervention was based [50]. Additionally, some patients receiving pulmonary rehabilitation had issues regarding transport to the facilities where the intervention was provided. Thus, these patients preferred receiving the intervention at home to reduce transportation time, illustrated by the following verbatim extract from one of the patients:

It’s (home early pulmonary rehabilitation) actually easier in many respects erm, than going into the, the COPD clinic (pulmonary rehabilitation venue) being one to one, but also it’s cutting down the amount of time of driving over there and all the rest of it [46].

The aspect of accessibility was viewed positively as care was provided at home in one of the early assisted discharge studies [50]. One patient described that privacy and being able to follow their own daily rhythm were advantages to accessibility. Conversely, disadvantages were breathlessness at night whilst being alone and being more able to cross physical limits [50].

#### Analytical theme 2: Aspects of interventions

Patients expressed positivity about different aspects of interventions, such as exercise reminders, medication reminders, and managing strategies [44, 51]. One patient’s description of medication reminders was: “It made such a difference to my life. I felt that it helped me regain independence and I was breathing better. I was using the preventer regularly and taking my medication.”[44] Additionally, patients did not recall the content of information leaflets because they were uninteresting [47].

Results from other studies showed that patients spoke positively about the intervention and the information received, as demonstrated by the following verbatim extract [45, 50]: “I am surprised that after 12 years having a lung disease I get breathing exercises for the first time” [50]. The information provided about COPD provided patients with a sense of control [45]. One patient realised that the intervention made them aware of their own mental health:

Yes, I think, possibly one thing came out of it on the psychological side. It asks ‘Do you often feel anxious or panicky?’ In general I would have said ‘no’, but I suddenly realised that ‘Yes, I do when I get breathless’ ... I hadn’t really thought about that before, so I could put that down and we could actually address that [45].

Patients had positive and negative perceptions regarding the use of robots in the care for COPD [44]. On one side, patients gave the robot a name and perceived it as a companion. On the other side, patients felt that the robot was useless, had difficulties interacting with the robot, or felt like they were being watched: “I felt like I was being policed because people were monitoring how much I was using my inhaler and I felt guilty or like I was being judged. It was an intrusion” [44]. Most patients using wearable technology found it easy to use. However, they thought a waistband used in one study was uncomfortable to wear and would prefer a wristband [47].

#### Analytical theme 3: Transition process from hospital to home

Patients experienced different issues regarding their hospital stay and the hospital staff [50, 52]. Patients complained about busy rooms and environment [50]; nurses and staff being too busy [50, 52]; problems with transport home from hospital [48]; and issues regarding their medicines: “Mistakes were made with the medicines” [50].

Some patients also expressed that they did not enjoy seeing different nurses and specialists [50], and that they wanted care from specialised nurses: “I think I’ll be quite happy and contented as long as I know I’ll be under the COPD nurses” [52]. However, patients also described being satisfied with the treatment in the hospital and at home: “Treatment in the hospital was good and the treatment at home was good as well” [50].

Patients with COPD experience difficulties adapting to life at home after hospitalisations due to exacerbations [48]. This was due to the abrupt change from being treated in the hospital to having to take care of themselves: “…And they send you home, and you come home, and you’ve got to start…erm, you gotta see to yourself” [48]. The patients in this study were recruited in an economically deprived area, which could mean that the patients had socioeconomic factors influencing the adaptation to life at home [48].

Patients in some of the studies were satisfied with receiving home visits from nurses, which made them feel safe and more confident, as illustrated by the following verbatim text extract from one patient [50, 51]: “It was safe, because I knew she was coming! If I did not feel 100% well, I knew that she was coming tomorrow to check me” [51]. The patients appreciated the attention they received from the visiting nurses and felt calm from their kindness [50].

#### Analytical theme 4: Individualisation of care

Patients preferred to have information individually adapted. Otherwise, the information was redundant or not relevant: “There is something about smoking on every page (of the brochure), but I have never smoked!”[51]

Pulmonary rehabilitation was preferred to be provided one-to-one in some cases, because the patients did not enjoy exercising in groups [46]. The patients were able to do the exercises in hospital, but it was too early after the exacerbation for some [46]. Similarly, patients in a different study felt too unwell to fully commit to the intervention and were overwhelmed with new appointments and medication after their exacerbation [47].

Additionally, patients were annoyed if the intervention interrupted other enjoyable activities. However, some patients adapted new routines because of the intervention: “It does give you a sense of purpose, you know, it goes off and you walk the dogs or go round to the neighbours or something like that. It clocks it up” [47].

## Discussion

More than 3,000 study reports were retrieved from our search, but only nine were included after the final screening. This indicates that there are only a few studies, which have reported qualitative data about interventions provided to patients with COPD. Furthermore, most of the included studies are from the UK. Only one of the included studies, Cox et al. (2018) [46], has used the Medical Research Council’s guidance for developing and evaluating complex interventions, which could suggest that more studies are required which have greater focus on intervention development and process evaluation. Cox et al. (2018) was the study that provided this review with the most diverse and detailed qualitative data, likely due to the process evaluation the authors undertook [46]. This reflects the importance of conducting process evaluations to inform future research. Whilst interventions to improve medicines management for people with COPD are commonly reported [10], we identified no process evaluations regarding this topic. Consequently, researchers are designing and delivering medicines-related interventions without a structured review of the process and recommendations of how it can be enhanced by future researchers.

Many different approaches for intervention delivery were used in the different studies we included. However, there were still some similarities between the studies. Two of the studies had an early discharge hospital-at-home service which had similar approaches to intervention delivery [50, 51]. In fact, only two of the studies had interventions in which the intervention was not provided fully or partially in the patients’ home [49, 52].

Qualitative data synthesised in this review found issues involving accessibility and delivery of interventions. Patients with COPD are a heterogenous group, as disease severity and symptoms vary between patients. This means that patients have different needs based on how their disease affects them. Thus, their needs should be considered when implementing interventions. Firstly, some patients may require additional help in terms of transport. Secondly, some patients require intervention delivery at different times, i.e., not too early in the morning. Lastly, some patients may require intervention delivery at home instead of at other facilities. The needs of patients are however dependent on context. Therefore, interventions should be adapted for local contexts and tailor the interventions for patients based on individual needs.

Technology provides solutions to different intervention delivery issues, such as with follow-up, education, and monitoring. However, many patients with COPD are older adults and may be unable to use technological devices. Additionally, more advanced devices may also make some patients feel uneasy due to their privacy being intruded—such as devices with cameras or microphones—which patients reported of in one of our included studies [44]. All interventions arguably need feasibility testing and would thereby expose issues related to acceptability and practicability. In the studies which included technology as part of the intervention, practical issues of technology use were among the most reported barriers for implementation. Many of these issues could also have been prevented by public and patient involvement throughout the process. Therefore, we recommend involving the public and patients from early stages of development and testing the feasibility of any developed intervention before feasibility testing.

Our findings show that close follow-up by healthcare professionals, such as nurses, is crucial in ensuring that patients can successfully adapt to their environment at home or community. This can be facilitated by hospital-at-home interventions. Two of the included studies had such a hospital-at-home approach, but almost all the included studies had an element of care being provided to the patient in their home. Even though hospital-at-home interventions may increase length of treatment, they also provide a lowered risk of hospital readmissions, lower depression and anxiety scores, increased quality of life, and cost savings [53, 54]. However, before any new interventions are introduced or changes are made to interventions, any issues regarding the transition process in itself should be addressed appropriately. Furthermore, it is important to recognise that patients have varying needs and living situations. Factors such as timing and location of the interventions may require careful individualisation.

Interventions should be individually adapted for each patient. This flexibility can help the transferability of interventions into different context, which is important for long term implementation as informed by the Medical Research Council’s guidance [13]. Future interventions should therefore include individually adapted elements to facilitate care. Some of these elements include self-management strategies, which have been previously recommended in literature [7]. Self-management strategies can help patients manage their own disease depending on what their individual needs are. As an example, information provided should be tailored and not given irrespective of needs, which is demonstrated by patients receiving information on smoking cessation even though they have never smoked [51]. Altogether, patients want and need interventions to be adapted to their situation. This requires the active involvement of patients in both the design process and provision of the intervention.

### Strengths and limitations

The screening process was undertaken by two independent researchers and the data extraction, analysis, and synthesis were double checked by another team member for agreement. This was done to increase credibility of our findings. There was high agreement between the two independent reviewers, with only minor discussions regarding which studies to include. A third researcher was included only after the screening process to discuss two of the studies, due to both screeners being uncertain whether to include them or not.

Our study was limited by language, as only studies in English or Scandinavian were eligible for inclusion, and we might have potentially missed some relevant studies in our screening process. Furthermore, our study may be affected by publication bias as we did not undertake an extensive search for grey literature, which is where process evaluations are frequently reported. Also, most of the studies are from the United Kingdom and other native English-speaking countries, which provides less data on different contexts and health care systems.

There are limitations to our systematic review because the qualitative data between the included studies varied greatly based on study design and qualitative analysis methods. In some of the studies—especially the clinical trials—the qualitative data lack details and the reporting is insufficient. Thus, we lack necessary information to fully interpret the collected data. Also, information about context is limited in the different studies, which further exacerbates the interpretation of the results.

It is noteworthy that medicines-related interventions were barely included in this systematic review, such as medicines reconciliation and medicines review [55, 56]. These interventions are frequently reported in literature and provide patients with medicines-related care and support, which is often a need for patients with COPD. Future research could benefit from including qualitative aspects from these interventions.

## Conclusion

Inaccessible interventions are unlikely to be effective. Therefore, public and patient involvement is required in the intervention design process and feasibility testing is needed once the intervention has been developed. Many different intervention elements were well received by patients. However, there was low acceptability and practicability regarding the use of technology. Transitioning from hospital to home is a difficult process for patients with COPD. Thus, it is important to address the issues regarding the transition process and optimise these before introducing new interventions or make changes to existing interventions. Additionally, future interventions should include individually adapted elements to facilitate a flexible approach to intervention delivery, especially regarding provided information. Researchers developing new interventions for patients with COPD should undertake process evaluations alongside studies for effectiveness and consider the local context of implementation sites for better engagement and adoption.

## Supplementary Information

**Additional file 1.**

**Additional file 2.**

**Additional file 3.**

**Additional file 4.**

## Data Availability

The datasets used and/or analysed during the current study are available from the corresponding author on reasonable request.

## Abbreviations

CASP: Critical Appraisal Skills Programme
CHF: Congestive Heart Failure
CINAHL: The Cumulative Index to Nursing and Allied Health Literature
COPD: Chronic Obstructive Pulmonary Disease
ESRC: Economic and Social Research Council
NHS: National Health Service
PRISMA: Preferred Reporting Items for Systematic Reviews and Meta-Analyses
PROSPERO: International Prospective Register of Systematic Reviews
RCT: Randomised Controlled Trial
