# Supplementary material for: Enhancing potential impact of hospital discharge interventions for patients with COPD: a qualitative systematic review
Source: BMC Health Serv Res. 2023 Jun 22;23:684. doi: 10.1186/s12913-023-09712-0 (PMC10288795; doi:10.1186/s12913-023-09712-0)
Supplement: Supplementary file 2 — Additional file 2. [file 12913_2023_9712_MOESM2_ESM.pdf]

Additional file 2

Search strategy

Due to the qualitative nature of this systematic review, the search strategy was based on Population, Interest, and Context (PICo). Related search terms including truncation was included for each subject. Keywords (MeSH terms for MEDLINE) was included in the search—where applicable—for each subject. For other databases, similar keywords to MeSH terms were used.

Table 1: Search terms based on population, interest, and context.

**Database**		**Population**	**Interest**	**Context**
MEDLINE (Through PubMed)	Free text<br>(Title/Abstract)	AECOPD<br>Chronic Obstructive Airway Disease<br>Chronic Obstructive Lung Disease<br>Chronic Obstructive Pulmonary Disease<br>COAD<br>COPD<br>Exacerbation	Admission*<br>Readmission*<br>Re-admission*<br>Discharge<br>Post-discharge<br>Transfer*<br><br>AND<br><br>Medicine*<br>Medication*	Hospital*<br>Ward*<br>Secondary Care<br>Tertiary Care
	Keywords<br>(MeSH terms)	Lung diseases, Obstructive<br>Pulmonary Disease, Chronic Obstructive	Patient Admission<br>Patient Discharge<br>Patient Readmission	Hospitals<br>Secondary Care<br>Tertiary Healthcare<br>Hospital Units
Embase (Through Ovid)	Keywords<br>(Title/Abstract)	Exp Chronic Obstructive Lung Disease/	Exp Hospital Discharge/<br>Exp Hospital Admission/<br>Exp Hospital Readmission/<br><br>AND<br><br>Exp Medicine/<br>Exp Medication/	Exp Hospital/<br>Exp Secondary Health Care/<br>Exp Tertiary Health Care/<br>Exp Hospital Subdivisions/
PsycInfo (Through Ovid)	Keywords	Exp Chronic Obstructive Pulmonary Disease/	Exp Hospital Discharge/<br>Exp Hospital Admission/<br>Exp Hospital Readmission/	Exp Hospitals/
CINAHL	Medical<br>Headings	Pulmonary Disease, Chronic Obstructive	Hospitalization<br>Patient Admission<br>Patient Discharge<br>Readmission	Hospitals<br>Secondary Health Care<br>Tertiary Health Care<br>Hospital Units
CENTRAL (trials)	Keywords	AECOPD<br>Chronic Obstructive Airway Disease<br>Chronic Obstructive Pulmonary Disease<br>COAD<br>COPD<br>Exacerbation	Admission*<br>Discharge<br>Post-discharge<br>Readmission*<br>Re-admission*<br>Transfer*<br><br>AND<br><br>Medicine*<br>Medication*	Hospital*<br>Secondary health care<br>Tertiary health care<br>Ward*
