# Supplementary material for: Enhancing potential impact of hospital discharge interventions for patients with COPD: a qualitative systematic review
Source: BMC Health Serv Res. 2023 Jun 22;23:684. doi: 10.1186/s12913-023-09712-0 (PMC10288795; doi:10.1186/s12913-023-09712-0)
Supplement: Supplementary file 3 — Additional file 3. [file 12913_2023_9712_MOESM3_ESM.pdf]

Additional file 3

Table 1: Extracted data on interventions based on the TIDieR checklist.

Reference	Name	Goals	Materials	Procedures	Provider	Delivery	Location	When and how much	Tailoring	Modifications	Fidelity strategies	Delivery as planned
**Broadbent**	iRobi robot COPD management	Reduce LOS, increase adherence, increase exercise, and improve QoL.	The iRobi Robot, Clinical COPD Questionnaire (CCQ), Smartinhalers, and instruction manuals for robot and inhalers	The robot had the following procedures installed:<br>(i) measure pulse oximetry; FEV; HR; and symptoms, mental state, and functional status using CCQ.<br>(ii) record adherence and give reminders<br>(iii) exercise reminders incl. instructional videos.<br>(iv) COPD education using video and pop-ups.<br>(v) allow patients to tell when they are not feeling well.<br>(vi) display health status and adherence trends to patients.	The robot was the provider of the intervention, but the robot was managed by two part-time physiotherapists through a web service.	The intervention was delivered through a robot in the homes of the patients. The intervention was delivered to patients with COPD post-discharge	Home-based intervention delivery in New Zealand.	The intervention was provided continuously for a period of four months. The usage of the robot differed from patient to patient based on their needs. The medication function was used on average 464 times per patient for the four-month period (range 41–1509). The exercise function was used 84 times on average (range 3–221). The measurement function was used 51 times on average (range 9–95). The entertainment function was used an average of 29 times (range 1–165). The education function was used 8 tines on average (range 0–77).	Not available.	Not available.	Adherence to the intervention was monitored by how many times the robot was used and which functions were used. If problems with adherence was detected by the physiotherapists, they called the patients to discuss concerns. If patients were not using the robots, they were encouraged through phone-calls.	Some technical issues were experienced. 50% of the robots experienced some type of technical issue. Technical issues included network connection issues, touch screen failures, hard disk failures, Smartinhalers not charging or connecting properly, and unplugging and driver issues with pulse oximeters.
**Buckingham**	HELPing older people with very severe COPD (HELP-COPD)	To help older people with very severe COPD	Questionnaires: FACIT, St. George's Respiratory Questionnaire, CAT, the hospital anxiety and depression scale, FACIT-Sp. Questionnaire about health care contacts.<br>Action checklist from HELP-COPD record.<br>Open questions	Baseline assessment by trial researcher was undertaken 2 weeks post-discharge (COPD-relevant history, smoking status, comorbidities) incl. Questionnaires.<br>Home visit by trial respiratory nurse 4 weeks post-discharge (discussion about	The main provider of the intervention was the trial respiratory nurse with experience in palliative aspects of respiratory care.	The intervention was delivered through home visits and telephone check-ups with the patients.	Recruitment was undertaken at Edinburgh Royal Infirmary or St John's Hospital, Livingstone in the UK. Intervention was given to patients in their homes, either by home visits	Patients were visited by the trial respiratory nurse 2-6 weeks post-discharge. Phone check-ups were undertaken at 1, 3, and 6 months.	The intervention was tailored to each patient depending on their needs. This tailoring was undertaken at the initial visits by the trial respiratory nurse using the HELP-COPD record that included open questions about concerns	Not available.	Telephone check-ups were used to check for intervention adherence and used as reminders to support the intervention.	Substantial attrition was experienced in the trial. This mainly related to severity of disease and bad timing of recruitment (just after exacerbation).

Additional file 3

Reference	Name	Goals	Materials	Procedures	Provider	Delivery	Location	When and how much	Tailoring	Modifications	Fidelity strategies	Delivery as planned
			guide from HELP-COPD record. Plastic folder and fridge magnet of the agreed action plan.	concerns and action plan was made). Patients were telephoned by respiratory nurse at 1, 3 and 5 months to check action plan progress. Trial questionnaires at 3 months (by post, incl. postal and telephone reminders). Trial questionnaires given directly by respiratory nurse at home visit at 6 months. Interviews shortly after HELP-COPD assessment to capture reflections, after 6 weeks to check for adherence to intervention, after 6 months to explore benefit and actions.			or by telephone.		and a checklist of agreed actions.			
**Clarke**	Early discharge service (EDS)	To reduce health care costs by moving care out of hospital	Not available	After discharge (at 3.5 days) patients received home-visits by nurses for 3 consecutive days up to 2 weeks. Home visits included clinical assessment and checking if medicines were taken appropriately.	The providers of the intervention were four nurses with experience in respiratory care based at a chest clinic attached to the acute care hospital.	Not available.	Not available.	Not available.	Not available.	Not available.	Not available.	Not available.
**Cox**	Early Pulmonary Rehabilitation (EPR)	To increase and maintain muscle force, strength, and size; maintain organ function; and improve psychological function	Hospital: Cycle ergometers ('bikes') were kept at participating sites. Instruction manuals were given to the physiotherapists. Home: Exercise manual	Hospital: Workload was set for the first session and two subsequent daily sessions. The workload was based on the maximum capacity of the participant. Following this	Hospital: A physiotherapist conducted the initial assessment each day to identify the workload required for the individual. Physiotherapists and	Hospital: The cycle ergometer was taken to the bedside by the physiotherapist delivering the session. Home: The intervention	Hospital: The intervention occurred at the patient's bedside (either in hospital or at home). The intervention was provided	Hospital: Patients completed 16 revolutions for both sets of limbs, three times a day for five consecutive days. Home: Four sessions	Hospital: The only adjustments that could be made by physiotherapists during the intervention involved adjusting the load to maximise the number of	Hospital: Patients who were discharged prior to five days were still included and received all usual care. The physiotherapist confirmed completion and	Physiotherapists at hospital EPR recorded number of rotations undertaken at sessions. Physiotherapists at home EPR recorded any adverse events that occurred and	Not available.

Additional file 3

Reference	Name	Goals	Materials	Procedures	Provider	Delivery	Location	When and how much	Tailoring	Modifications	Fidelity strategies	Delivery as planned
			and exercise diary was provided to all participants and physiotherapists.	assessment, patients completed 16 revolutions on the 'bike' with both upper and lower limbs. During the intervention heart rate, SpO_2 and symptoms of breathlessness or fatigue were monitored. Patients' oxygen was adjusted as required to maintain the SpO_2 within any prescribed target range. Home: 6MWD was made available to the physiotherapists, if not available they made their own assessment. Followingly, at the first visit the physiotherapists guided the participants during exercise using the exercise manual.	physiotherapist assistants could deliver the remaining sessions. Home: A senior physiotherapist conducted all the exercise sessions. The physiotherapist had a direct link to a hospital team in case of concerns about the participants.	was delivered by the physiotherapist at a suitable time for the patient (9–17, Monday to Friday).	in the UK. Home: The intervention was undertaken in the participants' homes.	were delivered over 2 weeks, starting within 72 hours of discharge. Visits could last from 20 minutes to 1 hour depending on participant ability.	repetitions undertaken while minimising symptoms of breathlessness or fatigue. Workload could be increased from session to session. Home: Physiotherapists could adapt the intervention to participants' capacity and limitations.	addressed any concerns. During the pilot the LOS was shorter than anticipated, so the eligibility criteria was changed to include patients with LOS<5d. Home: The intervention did not change during the trial.	any reasons for not taking part in a session.	
**Griffiths**	Patient-oriented discharge summary (PODS)	To provide discharge summary information for patients discharged from hospital.	Patient-oriented discharge summary (PODS)	The PODS form is filled out by the care team and given to the patient upon discharge.	Nurse navigator, resident physician, or other members of the care team.	The intervention was delivered by a printout of an electronic form to patients at discharge.	Canada. The intervention was given during the hospital stay and at discharge.	The information on the discharge summary was reviewed during the hospital stay, and later finalised at discharge and given to patient.	The information on the discharge summary was tailored to each patient.	Not available.	Not available.	Not available.
**Morton**	Admission and discharge care bundles	The admission bundle aims to reduce length of hospital stay and in-hospital mortality, whilst the discharge bundle aims to reduce re-admissions.	A 10-item list of interventions was used at intervention sites. Patients were given a written plan on how to manage additional AECOPD together with 'emergency' medicines on discharge.	The admission bundle consisted of: - Confirmation of a correct diagnosis of AECOPD. - Undertake an oxygen assessment and prescribe correct target range within 30 min. - To recognise and respond to	Information missing. Quality improvement strategies were encouraged in study sites using video-conferencing, face-to-face training sessions and one-to-one mentoring.	Intervention was delivered using a 10-item check list to patients hospitalised due to AECOPD.	The intervention was delivered in different hospitals in the UK (England and Wales)	Not available.	The intervention was tailored to individual participants by only providing appropriate elements from the checklist.	Not available.	Which elements were given to each patient were recorded.	Not available.

Additional file 3

Reference	Name	Goals	Materials	Procedures	Provider	Delivery	Location	When and how much	Tailoring	Modifications	Fidelity strategies	Delivery as planned
				respiratory acidosis within one hour of admission.<br>- Medication (steroids and nebulisers) to be administered within four hours of admission.<br>- Review by respiratory team to take place within 24 hours of admission.<br>The discharge bundle consisted of:<br>- Respiratory medication and inhaler technique assessment prior to discharge.<br>- Written management plan on AECOPD including 'emergency' medication prior to discharge.<br>- Assessment of smoking status and willingness to quit, including referrals to a stop smoking programme if wanted.<br>- Assessment of suitability for pulmonary rehabilitation prior to discharge.<br>- Organising a community follow-up within two weeks of discharge.								
**Orme**	Wearable and Mobile Technologies with Educational Support	To reduce sedentary behaviour at home in patients with COPD following hospitalisation due to AECOPD	Patient education booklet titled: "Sit Less, Move More, Live Healthier" which is adapted for COPD from "On Your Feet to Earn Your Seat". Inclinometer linked to a smart	Education arm: The intervention was delivered face-to-face in-hospital where written and verbal information was given via the booklet. The booklet contained seven main	The intervention was delivered by a researcher with a background in Physical Activity and Health.	The intervention was delivered by verbal and written education in-hospital. and with digital monitoring devices used in the home	The educational part of the intervention was given to patients in-hospital, whilst the monitoring was given at home for two	The educational part of the intervention was given once to patients in-hospital, whilst the monitoring was given continuously for two weeks following	The timings of the vibration prompts were determined by the patient in-hospital.	No changes were made to the intervention during the study period.	The educational part was audio recorded and later checked by two independent investigators. A checklist was used to determine which information was provided in the	Overall consistency of delivery was 77.3%. The quality of delivery was rated 0.2% as "poor", 9.4% as "good", and 90.4% as "excellent".

Additional file 3

Reference	Name	Goals	Materials	Procedures	Provider	Delivery	Location	When and how much	Tailoring	Modifications	Fidelity strategies	Delivery as planned
			device application with haptic feedback function.	suggestions: leave the house daily, make advertisement breaks active, stand-ups, tiptoe through the queue, increase steps, sit to stand with no hands, and treat the seat as a treat. Education and feedback arm: Real-time feedback on step count, sitting, standing, lying down, and sit-to-stand transitions. Haptic feedback was given as vibration prompts if prolonged sedentary time occurred.		setting. The intervention was given to patients following hospitalisation due to AECOPD.	weeks. The intervention was delivered in the UK.	hospital discharge.			education using a dichotomous scale (present or absent) and quality of delivery was evaluated as "poor, good, or excellent".	
**Utens**	Community-based hospital-at-home scheme (early assisted discharge)	Avoiding hospital admission, reduce length of stay, reduce pressure on hospital beds.	24-h telephone access to the hospital ward. Questionnaires at five different intervals.	The patients in the intervention group are discharged to home at day 4 of admission. Treatment is continued at home and supervised by nurses. The nurses have daily contact with the patients for 4 consecutive days, where they observe, counsel, and reassure patients. The nurses also help with issues regarding medication compliance, inhalation techniques, breathing- and coughing techniques, and diet. Two follow-up visits at the outpatient clinic to address issues and give questionnaires.	The providers are pulmonologists, general nurses, and pulmonary nurses. The general nurses conduct the home visits, while the pulmonologists and respiratory nurses conduct follow-ups. Initial screening of patients was undertaken by the research nurse and the pulmonologist.	The intervention was delivered by home visits and phone calls. The intervention was delivered to patients hospitalised for AECOPD.	The intervention was initiated in hospital during admission and continued at home from day 4–7. The intervention took place in the Netherlands.	The intervention lasted for seven days in total, where the first three days were in hospital and day 4–7 were at home for the intervention arm.	The intervention was tailored to each patient depending on their needs.	No changes were made to the intervention during the study period.	Not available.	Not available.

Additional file 3

Reference	Name	Goals	Materials	Procedures	Provider	Delivery	Location	When and how much	Tailoring	Modifications	Fidelity strategies	Delivery as planned
**Wang**	Early discharge Hospital-at-Home	(Reduce pressure on hospital beds, prevent readmission, reduce mortality)	No materials were used.	Evaluation of patient's clinical status, essential clinical parameters, obtained blood samples for later analysis when needed, and assessed whether patients could still be treated at home. After the consultation, decisions were made on frequency of follow-ups, therapy changes or hospital readmission. The patient and their spouse were invited for a dialogue about reflection.	The provider of the intervention was a specialised hospital nurse. The nurse could also consult a pulmonologist in case of worsening of patient's symptoms.	The intervention was delivered through home visits to patients recently hospitalised due to AECOPD.	The intervention occurred in patients' homes. The intervention took place in Norway.	Each visit lasted up to an hour daily over a period of three days. In the subsequent year, patients were offered three outpatient follow-up consultations with a pulmonologist at the hospital. The first visit was approximately six weeks after discharge.	The intervention was tailored to each patient by monitoring their clinical status and potential need for readmission to hospital.	No changes were made to the intervention during the study period.	Not available.	Not available.

AECOPD: Acute Exacerbation of Chronic Obstructive Pulmonary Disease, CAT: COPD Assessment Test, CCQ: Clinical COPD Questionnaire, COPD: Chronic Obstructive Pulmonary Disease, EDS: Early Discharge Service, EPR: Early Pulmonary Rehabilitation, FACIT: Functional Assessment of Chronic Illness Therapy, FEV: Forced Expiratory Volume, HR: Heart Rate, LOS: Length of Stay, PODS: Patient-Oriented Discharge Summary, UK: United Kingdom
