# Supplementary material for: Enhancing potential impact of hospital discharge interventions for patients with COPD: a qualitative systematic review
Source: BMC Health Serv Res. 2023 Jun 22;23:684. doi: 10.1186/s12913-023-09712-0 (PMC10288795; doi:10.1186/s12913-023-09712-0)
Supplement: Supplementary file 4 — Additional file 4. [file 12913_2023_9712_MOESM4_ESM.pdf]

Additional file 4

Table 1: Verbatim text extracts

Themes	Verbatim text extract
**Analytical theme 1: Accessibility and delivery of interventions**	
**Timing**	"Well to me, I could do it [hospital EPR] . . . I weren't fighting for breath and to me it was quite simple, quite easy . . . and I told . . . you know physio's like, he's say 'You alright?' and I say 'I could do another half dozen', kinda thing you know what I mean, said 'Well we'll come down in another 20 minutes have another go'. Which were fine, which were fine . . . might have been a little bit too easy."[1]
	"I tell them my legs were feeling a lot better, I could carry on and do more and they said 'No you've got to keep it to this, so many times' . . . But that were on that bike [hospital EPR]."[1]
	"Oh no, didn't push me hard, not hard just maybe a little bit too much in first I suppose it's like trial and error. They didn't push me, push me like go on make it thin ... I think he said I was 11 or something I don't know. They're nice people they're only trying to help I know they are."[1]
	"When you're in hospital there's no way you can exercise."[1]
	"[Home EPR was] quite straightforward yeah, it's only about 20 minutes, 25 minutes or something like that I do like y'know what I mean . . . that just does me like y'know what I mean."[1]
	"I'm still, I still find meself catching me breath and that."[1]
	"Just getting out of bed really, you know, I'm frightened of bringing that breathlessness on . . . I think, you know, the more I do and it's making me a bit more breathless, you don't know whether to stop or not."[1]
	"I think I do [worry about breathlessness when exercising] sometimes. I mean, I, I wish I could be different. I wish I could, you know, I, I want to get better and, and, and I know I will get better but I wanna be able to breathe a bit better."[1]
	"Oh they ask me while I'm doing the exercises do you want to rest, do you wanna give up, you can give up if you want and all this, that and the other. We're not pushing you it's you know."[1]
**Location**	«It would cost me 'bout £4/£5 in taxi, taxi fare . . . each way.»[1]
	«There's 13 steps to get up from my house so for transport . . . I've no chance you know what I mean . . . I'd have to go by ambulance, there's no way I can walk.»[1]
	«It [previous group PR] was good but it took up too much of my time . . . my husband used to take me up in the car. Now I can't go this time erm, apart from I've got a bad shoulder, I can't go it's too far, I can't get transport up there . . . I mean I wouldn't even know where to start because I can't get to the bus stop . . . because of pulmonary arterial hypotension I can't walk anywhere and I can't afford taxis up and down.»[1]
	«I don't like going out a lot . . . I've gotta be honest about that you know. I had to be pushed really to even to go to my daughter to be honest. I like my own company, since me wife died obviously and I like it in here."[1]
	«It's [home EPR] actually easier in many respects erm, than going into the, the COPD clinic [PR venue] being one to one, but also it's cutting down the amount of time of driving over there and all the rest of it.»[1]
	«If I'm being honest I'd like a bike in my house myself . . . And being able to go on it.»[1]
**Communication and information**	"The clear explanation about what they [staff] are going to do."[2]
	"The good explanation on the project."[2]
	"Insufficient preparation for going home, I expected more care at home with regard to medication and making coffee."[2]
	"They [hospital staff] promised more in the hospital."[2]

	"I am surprised that after 12 years having a lung disease I get breathing exercises for the first time" and "I am surprised to have learned the diagnosis COPD now and not earlier."[2]
**Access**	‘‘The first days I stayed not on the respiratory ward.’’[2]<br><br>‘‘I was not admitted to the respiratory ward, where I belong.’’[2]
**Flexibility**	"She always says that will that time be alright. It's always late afternoon…but she said if you wanna change it or anything, I said no whatever suits you I'm only sitting here you what I mean my social programme's not that good [both laugh] yeah."[1]<br><br>"Well, they asked me what time [to visit]… Yeah, they do it to like to what I asked them."[1]<br><br>"They come in the afternoon, it's roughly 2 o'clock…one of them must of got her in said 'you don't like morning ones?' I said 'I don't like if they're too early if they come about 11 or 12 o'clock if they can something like that'…once I've come round a bit."[1]<br><br>"in the class everybody were doing the same thing…you know and you can't say well you do that and I'll do that but they're all doing the same thing."[1]
**Introduction to intervention**	"Oh yeah, yes I really enjoy it as well as the benefits, I enjoy doing it yeah."[1]<br><br>"The bike was brilliant…and y'know with hurting my left hand shoulder it was bit stiff but y'know with doing that bike it, it seemed to cure it y'know what I mean…it's not too bad at all."[1]<br><br>"They said how important it is and all that."[1]<br><br>"I know nothing about it, I know nothing about it…Once I know that it entails I can make a decision then…I don't know enough about it love…I'm in the dark, I'm in the dark…I know nothing about it."[1]
**Distinctiveness to current services**	‘I’ve never had anything like this before. Not in-depth. You get asked basic questions [at the surgery]’[3]
**Analytical theme 2: Aspects of intervention**	
**Robots**	“Not useful. It didn’t do anything for me. I have been doing the same thing for years anyway.”[4]<br><br>“I didn’t need it. With this illness you never forget to take medication because otherwise you can’t breathe.”[4]<br>“I felt like I was being policed because people were monitoring how much I was using my inhaler and I felt guilty or like I was being judged. It was an intrusion.”[4]<br><br>“I felt like my privacy was invaded and I couldn’t go anywhere. I was worried about leaving it at home in case something went wrong or it was stolen.”[4]<br><br>“The robot would follow me around with its head. I hope that there was not a camera in it.”[4]<br>“It drove me batty. It always wanted me to do something.”[4]<br><br>“I couldn’t read it half of the time. I started off doing everything but I had problems going from screen to screen. It got very frustrating. It would tell me to take medications I had already taken.”[4]<br>“I named the robot after my great grandson because I miss him now that he is overseas. It made it like he is here with me.”[4]<br><br>“I will have no friend at home anymore! I liked having it in the house because it talked randomly and I would always touch it as I walked by.”[4]<br><br>“Bob (name of the robot) was like one of us. I would pat it on the head and he would respond. I often found myself having conversations with him.”[4]
**Questionnaires**	No quotes to underpin statement.
**Managing strategies**	“[The staff] was constantly dropping in to ask if I was thirsty or something. . .”[5]
**Medication reminders**	“It made such a difference to my life. I felt that it helped me regain independence and I was breathing better. I was using the preventer regularly and taking my medication.”[4]
**Exercise reminders**	“The reminders about the exercises were good, the robot would tell me to do it and my grandson would come and get me to say the robot needed me.”[4]

**Information leaflets**	"I read it [the leaflet]. Forgot it. It didn't really have anything in it that interested me, I don't watch much TV or anything."[6]
**Wearable technology**	"It [the device] was fine. No problem, fairly easy to use really. I put it on in the morning and left it on all day until I went to bed at night."[6]<br><br>"It's been a bit uncomfortable, because it's been hot, you know, and I couldn't put any thin trousers on because I'm wearing it, but it's been alright, yeah. Maybe a wrist thing would have been better for me."[6]
**Analytical theme 3: Transition process from hospital to home**	
**Efficiency of care and organisation**	"There is a shortage for staff. There is no time for the patient."[2]<br><br>"when you press the nursing alarm, you sometimes have to wait long for a response."[2]<br><br>"The mutual coordination [in the hospital] was lacking. This bothers me."[2]<br><br>"On the ward it was unstructured and disorganised."[2]<br><br>"The nurse of the home care organisation did not come. This should be better organised, especially during weekends."[2]<br><br>"Care should be tuned because of the medication and inhalations."[2]<br><br>"There was no clear information transfer to the respiratory nurse."[2]
**Life at home post-discharge**	"Might be boredom, just feel tired all the time. I just lay there. Now I just can't liven up. I don't make beds properly and I eat easy ready meals. Earlier on I could clean, make beds, cook a bit more. I can't go out alone".[7]<br><br>"They forget that when you're in hospital you're on oxygen all the time, you don't have to do a thing, you've got your food fetched to the bed, they help you shower. Everything's done...then suddenly a week later they say 'Oh you're fine now, your breathing's great.' Well of course it is, you've done nothing. And they send you home, and you come home and you've got to start...erm, you gotta see to yourself."[7]
**Specialist care and support**	"I think I'll be quite happy and contented as long as I know I'll be under the COPD nurses"[8]
**Staff perceptions**	"It means that they [patients] get the care they need, every time, it's always standard, it's always how they should be and we know it's always been done." (Lead Nurse, Acute Care)[8]<br><br>"With a care bundle, there is a better chance they are going to go out on the right treatment really, particularly if they have not been under the Respiratory Team, and they will have access to more services." (ED Consultant)[8]<br><br>"I think when patients get discharged I think our checklist that we have works really, really well because it's a good sort of pointer for us to try and get patients in to see the appropriate people." (Respiratory Nurse)[8]
**Courtesy and emotional support**	''I really appreciated the attention of the student nurse.''[2]<br><br>"The guidance at home gave me confidence."[2]<br><br>"Kindness, which makes me feel calm."[2]
**Discharge process in hospital**	"My son comes and gets me. I never bother with ambulances cos they take all day .. And, erm, I know they're busy, so my son has time off to come.and get me."[7]<br><br>Int: "How did you get back home?"<br>Patient: "Well stuck me in a wheelchair chucked me out the front and got a cab with the wife like."[7]<br><br>"There is always a four, five hour wait .[for medication]. [Sometimes]. I, I'll come home and whoever brought me home, me son or me daughter they'll go back [to the hospital] and get it."[7]
**Coordination of care**	No quotes available to underpin theme.

**Structure and facilities**	No quotes available to underpin theme.
**Issues with resources/staffing**	‘‘They [staff] have little time and therefore little attention.’’ (Patient in usual hospital care)[2]
**Technical quality**	‘‘Treatment in the hospital was good and the treatment at home was good as well.’’ [2]
	“Mistakes were made with the medicines.”[2]
	"The distribution of medicines was better last time [previous admission]."[2]
	"In the hospital the mouth piece of my inhaler was not cleaned." [2]
	"There was indistinctness concerning the medicines."[2]
**Family and friends**	“Well, [daughter] had a look at it and [son]. I have a son stays in the village as well and he had a look at it as well.”[3]
	“Everyone was interested in [the robot] when they came over!”[4]
	“If the family are too shy to ask me then they can look on the robot. The robot is not just for me.” [4]
**Feeling safe**	"It was safe, because I knew she was coming! If I did not feel 100% well, I knew that she was coming tomorrow to check me."[5]
	"The more you know, the safer you feel. You are not so frightened when you know what is what and get a proper explanation of this [disease]."[5]
**Analytical theme 4: Individualisation of care**	
**One-to-one therapy**	"It was a one-to-one approach which is where the recognition of the problem with the knees err, was appreciated because they could work a little bit closer with me to suggest alternatives.”[1]
	"I said no to that [group pulmonary rehabilitation] … because it was an 8-week course and I think no I didn't want to commit myself to anything like that. That's a group session I don't want that, I'm quite happy doing what I do in my own exercises.”[1]
**Individually adapted information**	"When you are at home you know what's what, and may think of various things that are relevant just then. It was just me and her and nothing to disturb us."[5]
	"She said that I do not need to get everything done in one day even if I am in a good period. I learned to distribute the energy evenly and listen to my body signals. She made suggestions and put me on the track to many good ideas.[5]
	"There is something about smoking on every page [of the brochure], but I have never smoked!"[5]
	"Is it possible to get any more information?"[5]
**Intervention fitting with routines**	“You feel it buzz on your back, so I just get up and walk in the kitchen or go and put the kettle on.”[6]
	“It does give you a sense of purpose, you know, it goes off and you walk the dogs or go round to the neighbours or something like that. It clocks it up.”[6]
	“I was annoyed that this thing was poking me in the back every half an hour, cause I didn’t want to move, I was watching something.”[6]
	“The first week was dreadful, I just wasn’t feeling myself. Anyway the next week I started feeling a lot better, started doing my normal things again.”[6]
**Patient-centred care**	"The care was personal."[2]
	"The care I received, problems were solved and the assistance of the staff."[2]
	"I was not treated and seen by my own pulmonologist."[2]
	"I saw different specialists." "I had to tell the same story over and over again."[2]
	"At home there were different nurses every time."[2]

**Individualised follow-up timeline**	No quotes to underpin theme.[9]
**Patient-carer relationship**	Community respiratory physiotherapist: «... giving someone ten, fifteen, twenty minutes, half an hour as a one-off is often not the way in which to address these sort of more complex patient problems that these patients present with, which is possibly why they've come up with nothing»[3]
**Being too unwell and overwhelmed**	"I've had to sit a lot. There's a lot going on with my health, and I just can't cope sometimes. I'm struggling with even my normal stuff."[6]<br><br>"You need so much energy to get through the day, it's difficult when you get home, and you're trying to recover and getting up is sort of difficult then, you just want to sit and relax and get better."[6]
**Health awareness**	«Yes, I think, possibly one thing came out of it on the psychological side. It asks 'Do you often feel anxious or panicky?' In general I would have said 'no', but I suddenly realised that 'Yes, I do when I get breathless' ... I hadn't really thought about that before, so I could put that down and we could actually address that.»[3]
**Disease information**	No quotes available to underpin theme.
**Self-triaging of symptoms**	"Well, I see how he is. I get that feeling that he needs to go to the hospital. I get a feeling of it, how he feels or how he looks. And usually, he doesn't like going to the hospital, I have to force him.» (son of patient with COPD)[9]

References:

1. Cox M, O'Connor C, Biggs K, Hind D, Bortolami O, Franklin M, et al. The feasibility of early pulmonary rehabilitation and activity after COPD exacerbations: external pilot randomised controlled trial, qualitative case study and exploratory economic evaluation. Health Technology Assessment. 2018;22(11):1-204.

2. Utens CMA, Goossens LMA, van Schayck OCP, Rutten-van Mölken MPMH, van Litsenburg W, Janssen A, et al. Patient preference and satisfaction in hospital-at-home and usual hospital care for COPD exacerbations: Results of a randomised controlled trial. International Journal of Nursing Studies. 2013;50(11):1537-49.

3. Buckingham S, Kendall M, Ferguson S, Macnee W, Sheikh A, White P, et al. HELPing older people with very severe chronic obstructive pulmonary disease (HELP-COPD): mixed-method feasibility pilot randomised controlled trial of a novel intervention. npj Primary Care Respiratory Medicine. 2015;25(1).

4. Broadbent E, Garrett J, Jepsen N, Li Ogilvie V, Ahn HS, Robinson H, et al. Using Robots at Home to Support Patients With Chronic Obstructive Pulmonary Disease: Pilot Randomized Controlled Trial. Journal of Medical Internet Research. 2018;20(2):e45.

5. Wang Y, Haugen T, Steihaug S, Werner A. Patients with acute exacerbation of chronic obstructive pulmonary disease feel safe when treated at home: a qualitative study. BMC Pulmonary Medicine. 2012;12(1):45.

6. Orme MW, Weedon AE, Saukko PM, Esliger DW, Morgan MD, Steiner MC, et al. Findings of the Chronic Obstructive Pulmonary Disease-Sitting and Exacerbations Trial (COPD-SEAT) in Reducing Sedentary Time Using Wearable and Mobile Technologies With Educational Support: Randomized Controlled Feasibility Trial. JMIR mHealth and uHealth. 2018;6(4):e84.

7. Clarke A, Sohanpal R, Wilson G, Taylor S. Patients' perceptions of early supported discharge for chronic obstructive pulmonary disease: a qualitative study. Quality and Safety in Health Care. 2010;19(2):95-8.

8. Morton K, Macneill S, Sanderson E, Dixon P, King A, Jenkins S, et al. Evaluation of 'care bundles' for patients with chronic obstructive pulmonary disease (COPD): a multisite study in the UK. BMJ Open Respiratory Research. 2019;6(1):e000425.

Additional file 4

9. Griffiths S, Stephen G, Kiran T, Okrainec K. “She knows me best”: a qualitative study of patient and caregiver views on the role of the primary care physician follow-up post-hospital discharge in individuals admitted with chronic obstructive pulmonary disease or congestive heart failure. BMC Family Practice. 2021;22(1).
